# Experiences of the Pharmacy-Led Weight Management Service: Views of Service Providers in England

**DOI:** 10.3390/pharmacy7030082

**Published:** 2019-07-03

**Authors:** Aliki Peletidi, Reem Kayyali

**Affiliations:** 1 Pharmacy Programme, School of Life Sciences, University of Nicosia, Nicosia CY-1700, Cyprus; 2 School of Life Sciences, Pharmacy and Chemistry, Kingston University, London KT1 2EE, UK

**Keywords:** pharmacy-led weight management service, experiences, pharmacists, pharmacy personnel, England

## Abstract

Obesity constitutes one of the main modifiable risks of developing cardiovascular disease. In the UK, in 2016, 30% of the adult population were obese (30% of females and 29% of males). Community pharmacies are ideally situated to offer weight management (WM) services, enabling individuals to control and lose their excess weight. This study aimed at exploring the views of the pharmacy-led WM service providers in England. Semi-structured interviews were conducted with 15 trained community pharmacists and pharmacy staff—11 (73.3%) from Kent, three (20%) from Kingston and Richmond and one (6.7%) from Hackney and City—offering the WM service, either owning or working in independent pharmacies or for pharmacy chains. All interviews were audio-recorded, transcribed and anonymised. The analysis was conducted using thematic analysis. Three themes emerged: training and support, barriers and approach. Interestingly, service providers (SP) stated that obesity is a tough topic to talk about: they found it difficult to start a conversation about it, even if they had received training to facilitate this role. Additionally, several barriers for running such a service were identified, such as lack of time, too much work pressure and too little advertising, which could potentially lead to poor sustainability of the service. SPs can effectively intervene in an individual’s weight through the WM service that they offer. It is clear that further training should be provided in order for SPs to feel more comfortable in approaching and communicating with people and to increase the public’s awareness of the pharmacy-led WM service, so as to ensure the service’s sustainability.

## 1. Introduction

Obesity is one of the main modifiable risk factors of cardiovascular disease (CVD). Unfortunately, the UK has high prevalence of obesity, leading the country to be dubbed as the “fat man of Europe” [1]. In 2016, 30% of the adult population were obese (30% of females and 29% of males) [2]. Weight loss is essential for individuals who are overweight or obese, since it bears physical, psychological and social health benefits. In 2007, the government-commissioned Foresight report determined that, if nothing is done about the issue, by 2050, 25% of children, 50% of women and 60% of men will become obese [3]. According to “Healthy Weight, Healthy Lives: A Toolkit for Developing Local Strategies” [4], community pharmacies are ideally situated to offer weight management (WM) services as part of a strategy for overweight/obesity reduction. 

In the UK, the WM service is included in the locally commissioned services (enhanced services) within the Community Pharmacy Contractual Framework (CPCF) [5,6]. However, in the UK, only a small number of studies exist in terms of the evaluation and success rate of pharmacy-led WM services. Most of the studies, even those with high dropout rates, concluded that pharmacy-led WM services can have a potential positive effect in weight-loss management and lifestyle alterations [7]. Boardman et al. [8] assessed the success rate of such services in supporting obese patients during weight loss. A total number of 281 individuals were enrolled; however, at 3 months, 110 remained in the programme and at 6 months, 59 remained. At 3 months, the main outcome was a mean weight reduction of −3.07 kg and a mean waist circumference decline of −3.87 cm. At 6 months, an additional weight and waist circumference decrease from baseline was observed with a mean change of −4.59 kg and −4.79 cm, respectively. Similarly, Morrison et al. [9] described the “Counterweight Programme” in community pharmacies in 2009. This took place in Scotland under the Keep Well scheme, in areas where the programme was not delivered by general practices. The programme offered patients advice, motivational support and education. Participants included had a BMI ≥ 30kg/m^2^ or ≥ 28 kg/m^2^ with co-morbidity, and a desire to lower their weight. The first meeting included a prescribed diet, including a calorie intake of around 500–600 Kcal/day. A total of 458 participants enrolled onto the programme, but only 314 remained enrolled for 12 months. At the 12-month time point, only 77 attended for measurements and had a mean weight reduction of 4.1kg with 32 (41.6%) achieving the target weight loss of ≥5%. Furthermore, Winter [10] and Bescoby et al. [11] conducted before and after studies (BAS) for weight reduction. In the Bescoby et al. study [11], pharmacists provided advice on healthy eating, exercise, food labels and guidance on how to set goals for losing weight. Participants had one-to-one appointments with pharmacists at weeks 1, 3, 5, 9, 13, 17, 21 and 25. Interestingly, only 10 participants (from the original 21 enrolled) remained in the study at 6 months and 60% of them (i.e. six patients) achieved weight loss of 5% or greater. Similarly, in the Winter study [10], only 10 participants achieved the pilot’s study aim of 5% weight reduction in the 12th week, and two participants reached a 10% weight reduction by the 24th week. Maximum weight loss was observed between weeks 1 to 8. Patients with a BMI of more than 35 kg/m^2^ achieved a low weight loss. Another published report, which showed positive results for a pharmacy-led WM programme, was the evaluation of the one-year Coventry PCT pharmacy WM service conducted in 2008. Overall, 160 participants were enrolled with a monitoring period of one year. Of those participants who finished the WM programme, 26.5% (9/34) achieved a weight loss of ≥ 5%. The mean weight loss was 3.7 kg (n = 34/160) [12]. 

When exploring the views of pharmacists on the delivery of WM services, the Krska et al. study [13] emphasised a lack of pro-active engagement on the part of pharmacists with their patients who were trying to lose weight. In addition, the same study assessed how the public generally viewed pharmacies as acceptable sites for WM services. This was a questionnaire-based study using two different questionnaires; one targeting the general public and the other for community pharmacists. The questionnaire for the community pharmacists established the frequency with which they counselled people who came to their pharmacies seeking help with WM issues. The public questionnaire included questions on whether participants had faced problems with being overweight or obese in the past, as well as how they perceived their weight as a whole. In addition, the questionnaire examined how receptive the public were about advice on weight loss; one of the options included was advice given by pharmacists, and participants were requested to list their first and last preferences. Krska et al. [13] highlighted that pharmacy was not seen as a setting of choice for WM services. The outcomes indicated that both females and males showed lack of awareness regarding the WM services that pharmacies provide. Another important finding of this study was that, for a number of people, their first choice in seeking advice for WM issues was not pharmacists, nurses or doctors. Instead, they preferred to go to gyms (36.7%, representing 65 responders). Additionally, Weidmann et al. [14] study investigated and identified the views and opinions of community pharmacists and their staff, including medicines counter assistants (MCAs), in relation to the pharmacy-led WM services that are offered. The study was conducted in the northeast of Scotland. Results indicated that pharmacists and pharmacy staff listed various advantages for offering a WM service through community pharmacies, such as a friendlier environment and better accessibility. On the other hand, some responses expressed concern that patients still prefer to turn to their doctors rather than pharmacists.

Interestingly, a recent review by Brown et al. [15] described pharmacy-based WM services as being as effective as other primary care strategies, and thus, they offer feasible options for WM. However, the Wright report [16] emphasised the findings of Jolly et al. [17], who noted that despite their feasibility, pharmacy-based services seem to be more expensive than commercial WM services and consequently less cost-effective. It is evident that apart from Weidmann et al. [14], the literature is lacking regarding the perceptions of service providers who run locally commissioned WM services in England, in relation to their views about the day to day running of the service and the training and resources required. Therefore, this study was designed to gather the experiences of English service providers offering pharmacy-led commissioned WM services, including their views and current role. Additionally, it aimed to recognise the training needs and development strategies in offering pharmacy-based WM services in order to implement such a service in a different European country. 

## 2. Materials and Methods 

The methodological approach of this study is reported based on the COnsolidated Criteria for REporting Qualitative Research (COREQ) checklist [18]. 

### 2.1. Design 

This study was based on exploratory descriptive qualitative research, using one-on-one semi-structured interviews to identify the views and the current role of service providers including pharmacists and pharmacy staff offering commissioned WM services. A qualitative research method was used, since it allowed for in-depth understanding of pharmacists’ and pharmacy staff experience in offering the WM service that a quantitative research approach cannot provide. This method is supported by the *phenomenology* [19,20] a philosophical approach intended to gain a comprehensive explanation of the phenomena under exploration from the participants’ point of view. 

The study was carried out between March and November 2016. All relevant documentation, including the interview schedule, was ethically approved in March 2016 by the Science Engineering and Computing (SEC) Ethics Committee (ID: 1415/032) in Kingston University London before data collection.

### 2.2. Study Setting and Sample

The study was conducted in England with trained pharmacists and pharmacy staff offering the WM service, either owning or working in independent pharmacies or for pharmacy chains. A purposive sampling strategy was used. A formal e-mail was sent initially by the first author (A.P.) to the service development pharmacist of the Pharmaceutical Services Negotiating Committee (PSNC) and then to all nine local pharmaceutical committees (LPCs) offering the locally commissioned WM service in England. Three LPCs replied positively to the e-mail, and as a result, interviews were conducted within pharmacies under the following LPCs: Kent, Kingston and Richmond and Hackney and City LPC. Reasons for not responding to the e-mail sent were not given. Potential participating service providers offering the WM service from these three LPCs who responded positively were contacted in person by the first author (A.P.) to inform them about the study and to encourage their participation. Furthermore, a participant’s information sheet (PIS) was given for further details. If a potential participant expressed an interest in taking part in the study, the first author (A.P.) then contacted them over the phone to schedule an appointment for the interview. Additionally, the sample size was not predetermined because with qualitative research there is no conventional guideline to determine precise sample size for interviews to be conducted. However, interviews were conducted until data saturation was achieved.

### 2.3. Data Collection

The interview schedule contained eight open-ended questions, mainly focused on WM. Pharmacists and pharmacy staff were asked to identify how they approach and intervene with overweight/obese patients, as well as to provide details on how they deliver the WM service. These included what measurements they take, what tools/equipment they use, the duration of the service, what techniques they use to help individuals to make a change in their lifestyle and what training they received to offer the service. Other questions addressed how they keep their knowledge up-to-date, and what they believe their strengths were in association with the offered WM service. Furthermore, a demographic information section was also included. Before the actual interviews took place, the interview schedule was piloted and validated by four pharmacists with no major changes.

Semi-structured interviews were conducted in person (face to face) by the first author (A.P.), who is a female registered pharmacist in the UK, Greece and Cyprus and has a PhD qualification. A.P. received training in conducting qualitative research during the academic year 2014–2015 at Kingston University. The interviews took place in each of the participating pharmacies’ consultation room for confidentiality purposes and to avoid any distraction. None of the researchers had any kind of relation with the interviewees before the study was conducted. Prior to the interviews, A.P. explained the reasons for doing this study. Written informed consent was also taken before each interview.

In total, 15 semi-structured interviews with trained pharmacists (n = 3) and pharmacy staff (n = 12) providing the WM service were conducted in England (14 females, 1 male). The interviews continued beyond the point at which thematic saturation was reached and no additional data could be received [21]. More specifically, thematic saturation was achieved after the 10^th^ interview. Nevertheless, interviews continued with all participants (additional five) who agreed to take part. The duration of each interview varied between 10 and 15 minutes, and all were digitally recorded after gaining consent from the participants and hand-written notes were taken during the interviews. Repeat interviews were not conducted with any of the participating pharmacists or pharmacy personnel. Before analysis, A.P. transcribed all the interviews verbatim, and anonymised them. 

### 2.4. Data Analysis

The interview transcripts were analysed thematically. The thematic analysis was performed inductively and deductively, and themes were extrapolated from the data obtained. Transcripts were not returned to participants for comments. A.P. read them independently, and listened to the recorded data, reading and re-reading a small number of interview transcripts and reviewing them manually, making notations directly onto the transcripts. The patterns within the responses in relation to pharmacists’/pharmacy personnel experiences in delivering WM service, their communication approach as well as their training were examined. The aforementioned enabled the identification of the primary emergent codes. The coding process was further developed after various rounds of reading the transcripts holistically, and discussion between the researchers which resulted in the grouping of the initial codes. The broad categories identified were: service providers’ views in delivering WM service and barriers. The remaining transcripts were read and re-read to ensure that all data had been coded. The coded transcripts were then checked and agreed by the co-author, RK. Each coded passage was grouped by thematic similarity by AP. This was followed by a discussion with RK and led to the development of common major themes. All themes were given an equal weighting within the thematic analysis. Agreement about all the emerging themes and subthemes was verified by both authors to ensure rigour, validity and trustworthiness of the themes presented and to ensure that there was no bias in data coding. R.K. has a Doctor of Philosophy (PhD) qualification. R.K. is a professor and a senior researcher in conducting qualitative research. The results are presented in the form of themes and subthemes. The themes are described by using illustrative quotes from each interviewee, who was then assigned a consecutive code (e.g., Great Britain Weight: GBW001, etc.). In the case of repetitive quotes found, the most characteristic quotes are presented.

## 3. Results

Personal and practice demographics are described in Table 1 below. The semi-structured interviews were taken from three different LPCs: 11 (73.3%) from Kent, three (20%) from Kingston and Richmond and one (6.7%) from Hackney and City. Participants were mostly female (n = 14, 93.3%) and were part of the pharmacy staff that was trained to offer the WM service. Pharmacists themselves offered the service in only three pharmacies (n = 3, 20%), one in Hackney and City LPC and two in Kingston & Richmond LPC. The minimum observed experience in the sample offering the WM scheme was 1 year, while the maximum was 8 years (n = 4).

Upon interviewing service providers, it was identified that the duration of the service varied between different LPCs, lasting anywhere between 10 weeks to 6 months. As interviews were taken from different LPCs, different WM schemes were described. In Kingston and Richmond LPC, they offered a 10-week programme called “Weigh2Go”; in Kent LPC, they offered a 12-week programme called “Fresh Start”; and in Hackney and City, they used to offer a 24-week programme.

All respondents pointed out that the WM service offered was through one-on-one, face-to-face appointments with an external referral for exercise if needed. In both Kingston and Richmond and Kent LPCs, the visits were weekly throughout the entire period. However, in Hackney and City LPC, the visits occurred weekly during the first 4 weeks, biweekly over the next 2 months, and monthly for the remaining 3 months. All visits took part in the consultation room in order to maintain privacy and confidentiality. In terms of how long they took with each patient, some service providers spent 15–20 minutes per session with each patient, while others tried to cover all the needs of their patients, spending an hour or more with them during each visit. However, some service providers added that during the first and second week, and during the last visit, they spent half an hour to an hour with each patient. Over the following weeks, shorter sessions took place, since service providers were already aware of what they needed to know about each patient.

In terms of tools and equipment that WM service providers had available, they mentioned that they had weight scales and height monitors, as well as machines that calculate Body Mass Index (BMI) and tape measures for measuring the waist. Pedometers were used in the past, but some clients forgot to return them, thus it was decided that they were not cost effective. Only one of the participating pharmacies still used pedometers during the service.

During a patient’s initial visit, service providers measured their height, weight and waist, and calculated their BMI. This was also repeated during the monitoring period. As well as from taking measurements from participants during each visit, pharmacy staff also discussed various lifestyle changes with them such as cutting down on portion sizes, giving them suggestions on eating out, and increasing physical activity. In addition, service providers provided participants with booklets containing useful information. The main ones used were from the British Heart Foundation (BHF). After the completion of the programme, service providers also offered additional weight measurements and advice, either for free or for a very small amount of money, e.g., one pound, according to some interviewees.

The thematic analysis identified three main themes in terms of implementing the WM service: knowledge, training and support, barriers and approach.

### 3.1. Knowledge, Training and Support

Almost all participants acknowledged the importance of WM and a balanced diet as part of CVD prevention. Positively, they all pointed out obesity as one of the main risk factors for CVD, underlining the significance of a balanced diet. They focused specifically on the importance of WM in combination with optimal cholesterol levels.

*“Primary prevention, I think weight loss [...] one of the biggest contributors to cardiovascular disease is obesity, so yeah.”*

(GBW003)

*“Obviously, if you have a problem with the cholesterol, you try to bring everything down in what you’re eating. Those’re the options. Yeah, and it’s all about lifestyle changes”.*

(GBW005)

Most respondents mentioned that prior to service delivery, they participated in a training course. Although the training varied between different LPCs, it lasted between 2–3 days. The training seminars included information on the service delivery, on how to approach and motivate clients (psychology of obesity), on how to measure them, as well as education on nutrition, exercise and obesity.

*“We originally went down to […] Council where they do a 2-day training session, and then they do a refresher every couple of years… It’s the go-to nutrition of people’s lifestyle, mindset, and actually how to read traffic lights and stuff like that.”*

(GBW006)

*“Okay, the course that we did was three days long, and it was quite intensive, and they take you through everything: how to screen somebody, how to... It wasn’t like a dietitian course, so it was mainly how to run the programme from start to finish. How to measure, how to approach healthy things… things which [are] high in calories, high in fat… meats as well as fats, like, some people you know believe that there is no sugar in one thing when actually there is a lot of sugar in it, so they teach you that as well, so it’s quite interesting for your own benefit.”*

(GBW002)

*“One day was food and diet, and the second day was motivational and behavioural…”*

(GBW004)

Another important type of support that was identified through interviews was the teamwork/support that pharmacies received through the service coordinators, as well as through the rest of the team. The support included help and advice when needed, all the necessary materials and handouts, such as leaflets, and having the coordinator at their disposal for any questions or problems they had.

*“I can call [the coordinator] anytime if I’ve got any question or queries, like... the paperwork got changed over and I wasn’t sure whether I had all the correct, up-to-date paperwork. But also, they order all of our leaflets and booklets and information through the British Heart Foundation.”*

(GBW011)

*“I’ve got everything: booklets, leaflets, you know they’re [the weight management team] quite supportive. They send a lot of things out and help with snack ideas and, yes, so generally they are quite there to ask questions.”*

(GBW005)

*“Okay, so the main coordinator […] she’s fantastic, she’s always there to help. If you’ve got any questions, any problems at all, she would always take you to the right team. Also, the pharmacist […] he’s great, you can always talk to him and he can put you straight with others other than him if you’ve got any problems.”*

(GBW012)

In order to keep their knowledge up-to-date, service providers attended refresher courses that programme organisers arrange for them once or twice a year. Service providers suggested that it would be beneficial if the refresher courses happened on a more regular basis. However, only one of them did not attend the refresher courses provided.

*“We have refresher courses that they provide, so we go along to them maybe once or twice a year. So we are always up-to-date. I am going on training.”*

(GBW002)

*“I think it would be really helpful if there was something like that [refresher training] on a regular basis, you could keep yourself up to date.”*

(GBW015)

*“There was a refresher in January I couldn’t go to, so the management are not very happy about it […] if anything is changed, obviously I’ll miss the refresher so there are refreshers over a couple of years.”*

(GBW001)

One of the three participating pharmacists shared that their staff needed more training so that they would be able to run the service by themselves. They also discussed the need to increase the number of pharmacies offering the service.

*“I personally think training is lacking in this, really lacking, and I think there is not enough people doing enough of this, and I think there is still a big gap for people and pharmacists can help skill them.”*

(GBW015)

However, service providers frequently read guidelines and professional journals/magazines and looked up information through the internet in order to keep themselves up-to-date in terms of the topic. On the other hand, one of the three participating pharmacists mentioned that they do their own Continuous Professional Development (CPD) as a way to keep their knowledge up-to-date.

*“I try to read. I try to read new guidelines, articles and listen.”*

(GBW003)

*“Okay, so I think the way to do it is making sure that you’re keeping up-to-date with the magazines that people publish from time to time.”*

(GBW015)

*“You can do your own CPD every year.”*

(GBW011)

### 3.2. Barriers

Several barriers for running the service were identified. These included: lack of time, work pressures and minimal advertising. These factors could potentially lead to poor sustainability of the service. In addition, appointments suffered due to time pressures. However, some of the respondents tried to find time for the service, as they emphasised that it is part of their role. Additionally, another barrier stated was the reluctance of patients to discuss obesity.

*“Sometimes, people don’t want to talk about their weight. They want to talk about anything else. So families, partners, pets, anything so they don’t have to talk about their weight, so you have to try and distract them from that, but that can be a barrier. […]”*

(GBW002)

*“There is lack of time, so it depends what’s a priority for you as a pharmacist? Reading prescriptions, is it providing services, or both?”*

(GBW015)

*“I have to make time; it’s my job to do it. So, I have to make time. People that come in to see me, they book in once a week, and I just have to make time to do it.”*

(GBW012)

Most of the interviewees appeared to be satisfied by their remuneration, while all agreed that there was a need for the service to be advertised more. In fact, the most prominent observation participants noted was that advertising was a big issue for the WM service. They expressed a need for more advertisement and more public awareness. As they mentioned, although it is a commissioned service, public engagement was a sustainability barrier for the service. If they did not gather enough participants, the NHS would discontinue the service. In addition, one participant reported that the current financial constraints that the NHS suffers from may reflect on the future of services and ability to advertise.

*“The problem is, if we don’t get enough people on a regular basis, then the NHS might decide to stop the service, because obviously they get paid for doing it, the people that run it, they get paid for doing it. So, if we don’t keep doing what we’re doing, to try and encourage people to keep coming in to join the scheme, then there is a possibility that it could stop.”*

(GBW004)

*“There […] is no funding right now so we just have to utilise the means we have and make most of the contact of the people we have with the patients because there is no funding anywhere, so we can’t say, Okay invest this money, invest in this because there is no money.”*

(GBW003)

*“Yeah, I don’t think that it is advertised enough. I think that actually, it should be more advertised on tellies—you know television and radios— […] like, January after Christmas, when people start looking at the television and they see, like, getting fit for the summer and it’s only about once a year, they tend to do that, you know. So if they actually, the NHS, maybe did more advertising on the radio or, you know, televised, you’d probably get a lot more people, you can actually get that support from this scheme because it’s a free programme, they don’t have to go to live streaming, they don’t have to pay money even like a one-to-one service. It’s like a one-to-one service, you know, and it’s free, and people will actually come to love it, you know, a lot of them aren’t aware of that”*

(GBW005)

*“[…] if there is more advertising, maybe, because most people come from the doctor. So maybe more advertising would be good, on TV or radio, something like that might be helpful.”*

(GBW009)

Another barrier that was identified was the service having such a long duration, and some service providers proposed a shorter duration as a way to avoid participants dropping out. This corresponded with interview responses highlighting that there was a decline in participation during the programme.

*“I think 10 weeks is too long for some people... 5–6 weeks, I think, is enough time for them to go off on their own and carry on, because sort of by week 7–8 they start to go back, or they don’t show up.”*

(GBW002)

*“Yes, after the fourth week [they may leave].”*

(GBW003)

### 3.3. Approach

Service providers shared that identifying and approaching overweight or obese people so that they could be included in the pharmacy-led WM programme was based on two factors: patient referral, or whether the patient decided to enrol in the programme on their own. They added that it was important to understand whether whoever enrolled was actually ready to make a change.

*“We don’t actually point them out, it’s something that people choose to do, so we’ve got posters up here. If people are concerned about their weight, they can ask about the […] service, which is a 12-week weight loss course. And unless it’s their decision, sorry, if it’s not their decision and they’re not ready to lose weight, then it’s not going to work.”*

(GBW004)

*“You can’t force people to choose to lose weight; it has to be their decision.”*

(GBW008)

Pharmacists and pharmacy staff do not play a proactive role in the WM service, but act on an unplanned case-by-case basis, using opportunistic approach. Typically, service providers rely on other services to attract people instead.

*“Sometimes when a pharmacist will be doing like Medicines Use Review (MUR ) or something like that, you know it’s the best way to bring that up with an overweight patient, and you know since we do a weight management scheme here and that’s what he normally does. It’s quite difficult, because if they are overweight, you can’t just say, ‘Hey, you know, you need to lose weight.’ So, you have to be very careful […]”*

(GBW002)

In addition, a pharmacist mentioned that they usually approach customers and initiate conversation when they come in to take prescribed or OTC WM medications, or if they are reading leaflets on slimming products.

*“Sometimes, people may be looking at some of the slimming products, so you can approach them and say, ‘Are you looking to lose weight? Did you know that we do a course here?’ and that’s the point where I initiate a conversation.”*

(GBW002)

*“When they give you a prescription, looking at the medication that they are taking and looking at, you know, if they are obese, then we sort of realise that there is a need to speak to this patient. […] What we do is, we say we do offer this service here, and that’s how we initiate. Obviously, you know, nobody will take it nicely if you say, ‘You know, I think you are a bit overweight.’ And we just say, ‘The medication you’re taking, you know if you want to lose weight, then we can help you do that, and we do run this clinic, you know, if you’d like to we can help you, and, you know, medication is so very good, but it all comes with a side effect, so you know, you have to think about it.’ ”*

(GBW003)

Service providers mentioned that obesity is a tough topic to talk about. They found it difficult to start a conversation about it, even if they received training to facilitate this role, as they felt that it is a very sensitive topic. Participants commented on their limitations in regard to identifying people who are obese or physically inactive. While most agreed that they did not feel very confident approaching people about their weight, they were comfortable talking about the service and promoting it.

*“I wouldn’t approach someone, if I’m honest. I wouldn’t approach someone and say, ‘You need to come on to [...]’ I guess I wouldn’t do that, if I’m honest. It is not something I feel comfortable with approaching people.”*

(GBW010)

*“I think if you’re going about it, it’s a difficult one, I mean, you can’t just walk up and say to someone, ‘You’re a bit fat,’ you know, ‘Do you want to do this [weight management programme]?’”*

(GBW011)

*“No, I don’t think I am confident in approaching people, because it is very difficult to say to somebody else who looks like [they] need to go on a weight manage programme”.*

(GBW012)

Some interviewees highlighted their personal experiences and how this shaped the way they act as healthcare professionals (HCPs), especially concerning this programme on WM. It was evident that service providers repeatedly utilised their own experiences in their discussions with patients so as to persuade them, using medical and dietary information along with statistics. While some tried to put into action what they had learned during the training on motivational interviews, their answers showed that their approach was not as structured as it could have been.

*“I wanted to make sure that, whatever I learnt from my personal experience... I wanted to make sure that every person that comes in my path... I will change the path and improve what they do, and that’s my ethos and hope, that really works for me.”*

(GBW015)

*“It’s something that I’ve done myself personally. I’ve lost a lot of weight through the same such as eating plan I offer here so it’s something I’m passionate about which gives me the motivation to help other people lose weight.*

(GBW004)

*“We talk. We show them statistics. We show them the risks, you know, by not losing weight, and you know there’s lots of statistics you can show people. […] Lifestyle’s restricted if you are very obese at a very young age. You see their lifestyle restricted, they become housebound. You could end up in a wheelchair. If you are diabetic, you could end up in amputation. So, we don’t try to scare them, you know, [but] we do talk about the risks if they do not lose the weight.”*

(GBW003)

Many respondents noted that providing evidence-based information and building good relationships with clients/patients allowed them to have a bigger impact on their clients’ health.

*“[…] I think I know my patient very well, and I know they’ll listen to me. I think they know if I say something, I’m not thinking from a business point of view because I’m trying to sell them something. They do know it’s because I care about them, that’s why I’m telling them, so I think they care. So, I think the trust of the patient in me is my biggest strength.”*

(GBW003)

*“I think my strength lies in communicating with other patients. I’ve built up a really good rapport with them and every person I see is completely different. I don’t generalise, I target each one individually. I talk about their needs, their wants, and it’s all about them, so they live here now and remember they might have taken in what you said, and it’s not just the blanket thing. And I think that really, really does help because every person is different.”*

(GBW012)

In addition, some respondents also referred to the privacy that they were able to provide to their clients and their personal experience with such topics as strength.

*“I think by having privacy, [...] and that’s very helpful because then they are very honest, they don’t feel like anyone is listening. They know it’s all confidential, it’s a lot of help as well, and it’s nice for them each time they come, that they are weighed every time on our scales, so it’s actually a routine and that’s helpful because I know how long the consultation will take, and they will know what’s happening each week.”*

(GBW002)

*“Because I can sympathise with them, because I’ve been there, I’ve done that, I’ve been overweight, I’ve lost weight. I know what it feels like.”*

(GBW004)

## 4. Discussion

The aim of this study was to interview English WM service providers; pharmacists and pharmacy staff to explore their views, as well as their knowledge and experience. Interestingly, the majority of the sample was female. This can be explained, as females have historically been attracted to pharmacy because it is widely perceived as a profession that gives them an opportunity to combine a professional career with a family creation [22]. Furthermore, according to the Global Pharmacy workforce and migration document, 63% of pharmacists are females in Europe versus 37% of male pharmacists [23]. 

According to Phelan et al. [24], it is the responsibility of HCPs to recognise modifiable risk factors associated with CVD, such as obesity, and to assist individuals in minimising their risks. However, despite the training received prior to service delivery, pharmacy-led WM service providers acted on an unplanned case-by-case basis in approaching people, since they did not find it easy to approach those with weight issues. Nevertheless, they recognised the need for the receiver of the service to be ready to make a change. Service providers enrolled patients who were either referred to the programme by other HCPs, such as doctors, or by other services such as NHS health checks, or even by self-referral. A reason behind this could be the fact that pharmacists and pharmacy staff reported that obesity is still a sensitive topic to discuss with their clients/patients. Obesity is a stigmatised health issue [24,25,26] and a very sensitive topic. This can explain the attitudes of service providers, who reported that they wait for clients to approach them on this subject first. Obesity stigma can lead to a vicious cycle as obese people can feel depressed and discriminated against, and often this leads to low self-confidence [24,27,28,29,30]. This puts a more prominent need for HCPs including pharmacists to have the confidence and skill to approach those with weight issues. As concerns have been raised that service providers are not trained enough in approaching and motivating people, further training should be offered. A possible solution to overcome this issue is to offer training such as “the stage-of-change model and motivational interviewing”, which is an effective training used in smoking cessation interventions. Capponetto et al. [31] outcomes indicated that training pharmacists, in the stage-of-change model and motivational interviewing, enhanced smoking reduction and cessation rates.

The participants agreed that, even though they try to keep their knowledge up-to-date, they still need refresher courses. This finding concurs with Weidmann et al. [14], who identified that inconsistent training should be addressed. The same study also suggested that training should focus on how to approach and assist participants to reduce their weight [14,32]. Consistent training with a focus on communication skills will help service providers feel more comfortable in delivering the service, as they admitted that they feel that they lack confidence and struggle to initiate discussions in relation to obesity due to its sensitivity as a topic. Such training should be expanded to all the pharmacy team. 

In fact, various studies ascertained that people do not feel comfortable talking to pharmacists and pharmacy staff about their weight, as they feel that pharmacists’ knowledge is lacking [33,34,35]. Nevertheless, the surveyed public in the Weidmann et al. study [33] agreed that participating in a WM service through pharmacies was more convenient for them than contacting a doctor, as they often face difficulties in booking appointments [33]. However, the interviewees mentioned that their appointments frequently suffered due to time pressures. On the other hand, the Jolly et al. [17] study, found that one of the reasons for a drop in public participation in the WM service was that participants found it difficult to arrange appointments with pharmacies, thus delaying weekly appointments. Another reason for drop outs mentioned in the above study could be the long duration of the service provided, which was highlighted as a barrier in this study.

A potential reason for people not discussing their weight with pharmacists and pharmacy staff is that they have expressed concern over the privacy that pharmacies offer [33,34,35]. This finding, however, contradicts the service providers’ views, who stated that privacy was one of the strengths that they offer. Another barrier is time pressure of delivering the service. This could be overcome by involving the whole pharmacy team in delivering the service. 

Even though the general public agreed in principle to the expansion of pharmacists’ roles [36], they seemed to be unaware of the public health services that pharmacists offer. Service providers mentioned the possibility of the service’s non-sustainability through pharmacies due to lack of advertising. They expressed the need for more advertisement to increase public awareness to ensure the sustainability of the service. These findings build on previous work, as described by Gidman et al. [37], Weidman et al. [14] and Eades et al. [38]. They concluded that members of the public are unaware of the WM services provided by pharmacies, probably due to the absence of advertising. However, as Weidmann et al. [33], Roth et al. [39] and Earle et al. [40] showed, the public was more aware of the BP monitoring and smoking cessation services, because they were successfully advertised through national campaigns. 

The NHS faces financial difficulties. These constraints may explain why pharmacists and pharmacy staff want to increase the public’s awareness about pharmacy-led WM services. The Robertson et al. report [41] explains how commissioning issues can influence patient care, due to stopping services or lowering their quality. According to the rapid report by Wright [16], as well as the systematic review by Brown et al. [15], clinical services, such as the pharmacy-led WM service, were found to be as equally effective as commercial weight loss programmes in reducing weight measurements by 3 to 5 kg in a short-term monitoring period (≤6 months). However, the cost of pharmacy-led WM services is higher than other commercial providers of such services. Therefore, due to financial circumstances, commissioners might eventually decide to discontinue the service, especially when a cheaper model can be equally as effective. This may explain why the interviewees expressed a fear of the WM service’s non-sustainability. 

One of the study’s limitations was that the service providers who were interviewed only represent three different LPCs in England. Additionally, as there were only three pharmacists (the remainder being pharmacy staff) and only one male, it seems possible that if a more diverse sample had been interviewed, a greater range of views might have been encountered and saturation might not appear to have been reached so soon. 

## 5. Conclusions

In conclusion, this study identified WM service providers’ experience and their views in delivering a pharmacy-led WM service. The findings suggest that further training should be provided in order for them to feel more comfortable in approaching and communicating with people, and that there is a need to increase public’s awareness about the pharmacy-led WM service, so as to ensure the service’s sustainability. In addition, the results provided a clear overview of the WM service, in particular the training that service providers should undertake before delivering the service, the structure of the WM service and the promotion required to ensure its uptake.

## Figures and Tables

**Table 1:** Personal Demographic Information UK Weight Management Service Interviewees (n = 15). LPCs: local pharmaceutical committees.

**Gender Distribution**		
**Sex**	**Frequency (n)**	**Percentage (%)**
Males	1	6.7
Females	14	93.3
**Age Distribution**		
**Age range**	**Frequency (n)**	**Percentage (%)**
18–24 years	2	13.3
25–34 years	3	20
35–44 years	2	13.3
45–54 years	6	40
55–69 years	2	13.3
**Years of Experience in the Weight Management Program**		
**Year range**	**Frequency (n)**	**Percentage (%)**
≤ 5 years	9	60
6–10 years	6	40
**Type of Pharmacies**		
**Type**	**Frequency (n)**	**Percentage (%)**
Chain	12	80
Independent	3	20
**Employment status**		
**Type**	**Frequency (n)**	**Percentage (%)**
Pharmacists	3	20
Pharmacy Staff	12	80
**LPCs**	**Frequency (n)**	**Percentage (%)**
Kingston and Richmond	3	6.7
Hackney and City	1	20
Kent	11	73.3
